# Effect of blister blight disease caused by *Exobasidium* on tea quality

**DOI:** 10.1016/j.fochx.2023.101077

**Published:** 2023-12-18

**Authors:** Yuxin Han, Xinyi Deng, Huarong Tong, Yingjuan Chen

**Affiliations:** Department of Tea Science, College of Food Science, Southwest University, Chongqing 400715, China

**Keywords:** Tea blister blight disease, *Exobasidium*, Microbial diversity, Soluble sugars, Tea quality

## Abstract

Blister blight, as one of the most threatening and damaging disease worldwide, mainly infects young organs and tissues seriously affecting tea growth and quality. In this study, the spread of pathogen on tea leaves were examined by toluidine blue staining, scanning electron microscope and transmission electron microscope analysis. The composition and abundance of fungal community on leaf tissues were firstly analyzed. Sensory evaluation and metabolites analysis indicated that diseased tea leaves had strong sweet taste and soluble sugars contributed significantly to the taste, while metabolites showing bitter and astringent taste (caffeine, catechins) were significantly decreased. According to the biological functions of differential metabolites, sugars including 7 monosaccharides (d-xylose, d-arabinose, d-mannose, d-glucuronic acid, glucose, d-galactose and d-fructose), 2 disaccharide (sucrose and maltose) and 1 trisaccharide (raffinose) were the main differential sugars increased in content (>2 fold change), which was of great significance to sweet taste of diseased tea.

## Highlights

• Spread and development of blister blight disease on tea leaves were fully described.
• Composition and abundance of fungal community on infected tea leaves were revealed.
• Soluble sugars significantly contributed to the enhanced sweetness of diseased tea leaves.
• Monosaccharides were the main differential sugars significantly induced in infected tea leaves.

## Introduction

Tea, as a beverage, is popular for its healthy benefits and pleasant flavor. Tea plant is a perennial economic crop which is susceptible to various kinds of destructive foliar diseases due to the warm and humid growth environment in tea plantations that provides a suitable microclimate for the breeding of pathogens (Baby, 2002). Among these diseases, blister blight caused by *Exobasidium vexans* Massee is considered as the most serious disease in the world that mainly damages tender leaves, buds and young fruits, resulting in yield loss and quality decrease (Punyasiri et al., 2005, Sen et al., 2020). At the tea plant experimental plots of Sri Lanka, the disease first occurs through the formation of appressoria and penetration of the cuticle, after penetration visible translucent spots are formed, and with the development of spots, characteristic circular blisters appear on the surface of young tissues, finally resulting in the infected tea leaves distorted and infected stems broken off at the point of infection (Punyasiri et al., 2005). Blister blight disease occurring in almost all the tea plantation regions of Asia, has become the major problem on tea plants that causes serious economic losses (Sinniah et al., 2016, Baby, 2002, Guo et al., 2005). It is reported that blister blight disease is more serious in alpine tea gardens in southwest and south of China. In epidemic years, the incidence of blister blight in tea growing regions located in southwest of China can reach 40–50 %, and even reach 90 % in severe cases (Chen and Sun, 2013, Sen et al., 2020). It was estimated to cause about 33 % crop loss in Sri Lanka (De Silva et al., 1992), 25 % in Indonesia and as high as 50 % in South India, respectively, in tea fields in the absence of control measures (Radhakrishnan and Baby, 2004). Besides yield loss, blister blight disease negatively affects the growth of tea trees, resulting in the quality decrease of processed tea which is even exceeded the economic threshold level 35 % (Ponmurugan et al., 2016, Radhakrishnan and Baby, 2004).

Tea has abundant non-volatile and volatile metabolites such as tea polyphenols, amino acids components, caffeine, soluble sugars, lipids, and aromatic compounds, which are proved to be closely associated with the flavor and quality of tea (Cao et al., 2021). Some quality related metabolites such as caffeine, epicatechin (EC) and theanine (Thea) also have been reported to have potential effect against various kinds of viral or fungal diseases (Sharma et al., 2021, Punyasiri et al., 2005). The development of pathogen in various stages showed different effect on pathogenesis related protein, anti-oxidative enzymes and flavonoid pathway in tea, suggesting the possible role of some chemical compounds (i.e., reactive oxygen species, anthocyanins, lignins, catechins) and other synthesized compounds in acting as antifungal agents in different tea cultivars (Nisha et al., 2018). A large amount of studies have validated that various kinds of biotic (ie, disease infection, insect attack) and abiotic (ie, mechanical damage, environment conditions) stresses can remarkably influence the quality and flavor of tea by significantly changing the composition and amount of metabolites, especially affect the aromatic compounds in tea plants (Chen et al., 2017, Cho et al., 2017, Mei et al., 2017, Zeng et al., 2019). Tea processed from blister blight infected fresh tea leaves is fragile and shows obvious bitter taste, and the level of chemical components related to tea quality has decreased significantly, especially tea polyphenols and catechins (Guo et al., 2005, Jayaswall et al., 2016). Premkumar et al. (2008) reported that after the infection of blister blight disease, there were remarkable reduction in the amount of tea polyphenols, catechins, sugar, nitrogen, proteins, amino acids and other substances in infected tea leaves. Mur et al. (2015) analyzed the chemical compounds including caffeine, flavan-3-ol, flavone and flavonol in blister blight infected tender tea leaves by using high performance liquid chromatography with online photodiode array detection and electrospray ionization-tandem mass spectrometry (HPLC-PDA-ESI/MS), and found that kaempferol and quercetin glucosides, kaempferol triglycosides and some catechin-class antioxidants were increased, while the level of caffeine and apigenin and myricetin glycosides were remarkably reduced as disease progressed. However, limited information are still available on the tea quality affected by blister blight disease.

In this study, the development and spread of blister blight on tea leaves, and the composition and abundance of fungal community on leaf tissues were fully investigated. After the infection of the disease, major metabolite differences in healthy and diseased tea leaves were studied, centring on the metabolites that contributed to the tea taste, which is helpful to improve the understanding on the influence of blister blight on tea quality.

## Materials and methods

### Materials

During 2018 to 2020, tea leaves displaying blister blight symptoms were observed in high mountain tea plantations above 1000 m altitude located in Quxian county, Dazhou city, Sichuan province, China (30°85′N, 106°94′E). The typical blister blight symptoms were collected to show the symptom development. To analyze the characteristics of the blister blight disease, more than 100 fresh diseased tea shoots showing blister blight symptoms were harvested at April 2020, and used for microbial diversity analysis and microscopic analysis of pathogens including tissue section observation, transmission electron microscopy (TEM) and scanning electron microscopy (SEM) analysis. To investigate the effect of blister blight on tea quality and flavor, diseased tea shoots with one bud and two leaves (500 g) were sampled, while healthy tea leaves were used as a control. All the experiment had three replicates. Tea samples used for metabolites profile analysis were fixed by microwave (2–3 min), dried in a tea dryer machine (1 h, 80℃), finally milled and stored in a − 80℃ freezer.

### Microscopic analysis

#### Toluidine blue staining (TBS)

Tea leaf tissues (1 × 1 cm) were firstly fixed with FAA (Formaldehyde-acetic acid–ethanol) (Solarbio, Beijing, China), then dehydrated with ethanol, embedded in paraffin and routinely sliced. The slices were placed in xylene I (Sinaopharm Group Chemical Reagent Co. LTD) for 20 min, xylene II (Sinaopharm Group Chemical Reagent Co. LTD) for 20 min, anhydrous ethanol I (Sinaopharm Group Chemical Reagent Co. LTD) for 5 min, anhydrous ethanol II (Sinaopharm Group Chemical Reagent Co. LTD) for 5 min, 75 % alcohol for 5 min, and washed with double distilled water (ddH_2_O). The dehydrated slices were stained in toluidine blue solution (Wuhan Google Biotechnology Co. LTD) for 6 min, then washed with ddH_2_O, finally the qualified slices were dried in an oven. Transparent sealing: the dried slices were treated in xylene (Sinaopharm Group Chemical Reagent Co. LTD) for 5 min, then taken out to dry, finally sealed with neutral gum for microscopic examination.

#### Scanning electron microscopy (SEM)

Tea leaf tissue blocks (3 mm^2^) were harvested within 3 min and washed with phosphate buffer saline (PBS) (Servicebio, Wuhan, China) gently, then immediately immersed in electron microscopy fixative (Servicebio, Wuhan, China) at room temperature for 2 h, finally transferred into 4 °C for preservation. After fixation, tissue blocks were rinsed with 0.1 M phosphate buffer (PB, pH 7.4) for 3 times (15 min each), transferred into 1 % osmic acid (Ted Pella Inc.) for 1–2 h at room temperature, then washed in 0.1 M PB (pH 7.4) for 3 times (15 min each). Leaf tissue blocks were dehydrated with different ethanol concentrations (30 %, 50 %, 70 %, 80 %, 90 %, 95 %, 100 % and 100 %) in sequence for 15 min each, finally were placed in isoamyl acetate (Sinaopharm Group Chemical Reagent Co. LTD) for 15 min. After 0.1 M PB (pH 7.4) wash, the samples were dried with critical point dryer (Quorum, United Kingdom), then were attached to metallic stubs by using carbon stickers and sputter-coated with gold for 30 s, finally were observed with SEM (Hitachi, Japan).

#### Transmission electron microscope (TEM)

Sampled fresh tea leaves were cut into 1 mm^3^ tissue blocks within 3 min, then quickly fixed in the electron microscopy fixative (Servicebio, Wuhan, China) at room temperature for 2 h, finally stored at 4 ℃. After fixation, leaf tissue blocks were rinsed with 0.1 M PB (pH 7.4) for 3 times (15 min each). Post-fixation: tissue blocks were fixed in 1 % osmic acid (Ted Pella Inc.) that prepared in 0.1 M PB (pH 7.4) at room temperature for 7 h away from light, then washed three times with 0.1 M PB (pH 7.4), 15 min each time. Tissue dehydration: treated tissues were dehydrated in 30 %, 50 %, 70 %, 80 %, 95 %, 100 % alcohol in sequence, 1 h each time, and then in ethanol: acetone = 3: 1, ethanol: acetone = 1: 1 and ethanol: acetone = 1: 3, for 0.5 h each, respectively, finally in pure acetone for 1 h. EMBed 812 (SPI, USA) was used for osmotic embedding, then the embedded plates were placed in 65℃ oven to polymerize for 48 h. Treated blocks were cut into thin slices (60–80 nm) with ultra-microtome (Leica, Germany). The copper mesh slices were dyed in the dark with uranium acetate saturated alcohol solution (2 %) for 8 min, rinsed in ethanol (70 %) for 3 times and in ddH_2_O for 3 times, dyed in lead citrate solution (2.6 %, without CO_2_) for 8 min, washed with ddH_2_O for 3 times, finally put into a copper mesh box to dry overnight at room temperature. The prepared slices were observed with TEM (Hitachi, Japan).

#### Microscopic examination

Diseased tea leaves exhibiting blister blight with abundant basidiospore were observed with a U-TV0.5XC-3 microscope (Olympus, Japan). The shapes and sizes of basidiospore were recorded by measuring at least 30 randomly selected basidiospore.

### Microbial diversity analysis

To analysis the composition of microbial communities in diseased lesion tissues showing blister blight in Quxian, Sichuan province, China, genomic DNA of lesion tissues was extracted by using HiPure Soil DNA Kits (Magen, Guangzhou, China) based on the manufacturer’s protocols. ITS gene region was used for the identification of fungal communities. Gene region of ITS2 was amplified with primer pair ITS3_KYO2 (5′-GATGAAGAACGYAGYRAA-3′) and ITS4 (5′-TCCTCCGCTTATTGATATGC-3′). The amplified products were purified and then quantified using Nanodrop procedures. Sequencing was conducted by Genedenovo Inc. (Guangzhou, China) by using Illumina Hiseq 2500 PE250 platform (Illumina, San Diego, CA, USA). The α-diversity indices such as Chao1, Simpson, and Shannon were quantified to show the fungal diversity in diseased tea leaves according to the Operational Taxonomic Units (OTUs) richness in QIIME (version 1.9.1) (Caporaso et al., 2010). The species distribution river map of the fungal composition was plotted using R project ggplot2 package (version 2.2.1). The heatmap of species abundance was constructed based on pheatmap package (version 1.0.12) in R project. Functional group (guild) of the microbial communities were inferred by FUNGuild (version 1.0). The phylogenetic tree was constructed by Neighbor-Joining (NJ) method in MEGA6. The distances of evolutionary were computed by the method of Maximum Composite Likelihood.

### Sensory evaluation

Quantitative descriptive analysis was conducted by a sensory panel with seven well-trained panelists (4 males and 3 females, 40–55 years old) from the Department of Tea Science, Southwest University, China. All the panelists had been informed of the sensory evaluation, and agreed to take part in this research and use their information. The rights and privacy of all the panelists were protected during the research, and ethical approval for the involvement of human subjects in this study was granted by Southwest university research ethics committee. Total 3.0 g tea sample was weighed, and brewed with 150 mL boiling water for 5 min, then tea infusion was used for taste evaluation. Taste evaluation included the common characteristics such as bitterness, astringency, umami and sweetness (Cao et al., 2021). Sensory evaluation was conducted by the national sensory evaluation method for tea GB/T 23776–2018 (Gong et al., 2018). Values of taste attributes were scored by a 10-point scale and 5 intensity were evaluated in given tea samples: 0–2 represents extremely weak, 2–4 represents weak, 5–6 represents moderate, 7–8 represents strong, and 9–10 represents extremely strong. The highest and lowest scores for each attribute were removed and the mean value was used for the evaluation.

### Sugars analysis by GC–MS

The content and composition of sugars were analyzed using gas chromatography-mass spectrometry (GC/MS) (Chen et al., 2023). Tea sample (20 mg) was mixed with 500 μL methanol: isopropanol: water (3: 3: 2, V/V/V) solution, vortexed for 3 min, ultrasound for 30 min, and then centrifuged at 12,210 g (3 min, 4℃). Agilent 8890 gas chromatograph coupled to a 5977B mass spectrometer with a DB-5MS column (J&W Scientific, USA) was used for GC/MS analysis. Helium was the carrier gas with a flow rate of 1 mL/min. Injections were made with a split ratio 5:1 and the total injection volume for each sample was 1 μL. The oven temperature was set at 170℃ for 2 min, then increased to 240℃ at 10 ℃/min, finally raised to 280 °C at 5 °C/min, raised to 310 °C at 25 °C/min and kept for 4 min. Selective ion monitoring mode was used for sample analysis. The orthogonal projections to latent structure-discriminant analysis (OPLS-DA) were applied to distinguish the significantly differential metabolites between different tea samples. Significantly changed metabolites between healthy and diseased tea leaves were determined by absolute Log2FC (fold change), variable importance in project (VIP) and P value (Student’s *t* test). When the fold change ≥ 2 or ≤ 0.5, P value ≤ 0.5 and VIP ≥ 1 between two groups, the metabolites difference was considered significant. The metabolites annotated in Kyoto Encyclopedia of Genes and Genomes (KEGG) compound database (https://www.kegg.jp/kegg/compound/), were then mapped into metabolic pathway based on database search (https://www.kegg.jp/kegg/pathway.html). The P value represented the metabolites enrichment in a pathway, and P ≤ 0.05 was indicative of significant enrichment.

### Catechins, caffeine and amino acids analysis by HPLC

Catechins and caffeine contents were analyzed by using high performance liquid chromatography (HPLC) (Agilent 1200, Agilent Technologies, Santa Clara, CA, USA) (Chen et al., 2023). Prepared tea infusions were analyzed by an Agilent Eclipse XD8 C18 column (250 mm × 4.6 mm i.d., 5 μm; Thermo Electron Corporation, Waltham, MA, USA). The volume of injected sample was 5 μL with 0.9 mL/min flow rate. The wavelength of UV detection was 278 nm. The composition and content of amino acids were analyzed with HPLC system (Chen et al., 2023). The HPLC conditions were as follows: mobile phase A was 4 mM sodium acetate (pH = 5.5); mobile phase B was 80 % acetonitrile solution; column temperature was 35 °C; flow rate was set at 0.9 mL/min. The wavelength of UV detection was 360 nm. The results were expressed as mean value ± standard deviation (SD). The *t* test of independent sample was used to calculate the statistically significant difference between different groups (P ≤ 0.05) by IBM SPSS Statistics 20.0 (SPSS Inc., Chicago, USA).

## Results and discussions

### Disease symptoms on tea caused by blister blight disease

**Fig. 1:**
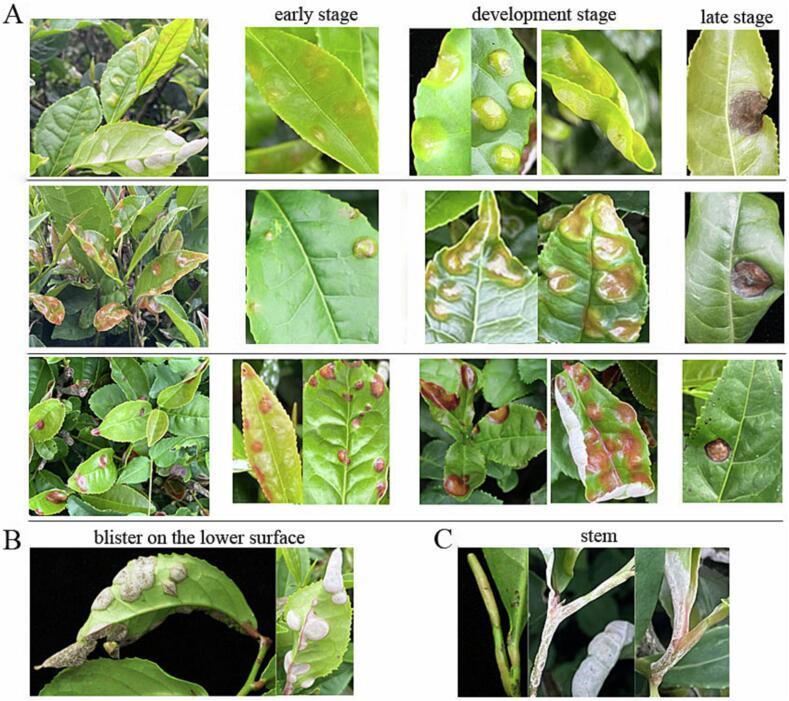
Symptoms of blister blight disease on tea plants. (A) Tea leaves infected with blister blight disease at different stages. (B) Blisters on lower surface of tea leaf. (C) Blisters on stems.

As an important tea foliar disease, blister blight mainly infected young organs and tissues including tender leaves, buds, petioles and stems (Fig. 1), directly affecting tea industry qualitatively and quantitatively. The damage to tender shoots and leaves was the most serious, while mature leaves and stems were not susceptible to blister blight disease. The symptoms caused by blister blight were firstly described in details by Petch (1923). Blister blight usually has a short life cycle of 11–28 days due to the different climatic conditions in different locations (Sen et al., 2020). In this study, the initial symptoms of blister blight disease usually infected tender leaves that caused light green, yellow-green, light yellow or slightly red tiny water-soaked translucent spots after 5–15 days of infection, then the spots gradually developed into well-defined circular and larger lesions with the diameter of 1–13 mm (Fig. 1A). Simultaneously, the spots were sunken into slightly concaves, and on the downside, they bulged correspondingly, finally resulting in typical blister lesions, but there were also a few lesions that bulged towards the upper of the leaves (Fig. 1A). The concave upper surface of the blister was smooth and shiny, and the convex surface was generally thickened with powdery coating, then become pure white velvety due to the intensive growth of basidiospores (Fig. 1B). At the most serious stage, the adjacent blisters might fuse into large irregular lesions, and diseased tender shoots became distorted or irregularly rolled. At late stage, the mature basidiospores were released and spread by airflow, and the blisters turned dark brown or purple-red ulcer-like and dried up, or even form holes (Fig. 1A), and might be infected by other saprophytic fungi. When young stems and petioles were infected, the diseased tissues showed obvious swelling, which turned to dark brown withered spots in the later stage (Fig. 1C). The damage to stems was more serious, as the infected stems bent over and broke off at the affected spot, affecting the growth of tea plants.

Blister blight is a low temperature and high humidity type disease, and the mycelium is latent in the living tissue of the diseased leaves for over-wintering and over-summering. When the climatic conditions were suitable, the basidiospores fell to the water droplets on the young leaves or new shoots of the tea trees with the wind, germinated and invaded the tea leaves in the water environment, and then the basidiospores reproduced in large numbers, so repeatedly infected, thus causing large yield and economic loss (Sinniah et al., 2016, Mur et al., 2015, Sen et al., 2020). It has been reported that a white powder coating on blister can produce two million spores in 24 h which represents an enormous consumption of metabolites and energy of tea (Baby, 2002). In Quxian county, Sichuan province of China, blister blight disease mainly occurred in high mountain tea plantations above 1000 m altitude, infection began to appear when the temperature was above 13℃, and with the increase of temperature and humidity, the disease aggravated. Higher temperature coupled with less rainfall and low humidity during August in summer resulted in the disappearance of blister blight disease, and during December to January in winter, blister blight disease also disappeared when the temperature was lower than 12℃ (Table S1). As shown in Table S1, In the main tea growing areas worldwide, the occurrence period and damage degree of blister blight was closely associated with the different climatic conditions, therefore, the disease incidence were remarkably different in different tea plantations such as Sichuan, Guizhou, Anhui, Yunan, Zhejiang and Hunan province of China, and Sri Lanka, India, etc (Wang et al., 2013, Liu et al., 2021, Liu, 2017, Shi et al., 2016, Sinniah et al., 2016, Mur et al., 2015, Ran et al., 2021). The relationships between rainfall, temperature and humidity to the intensity of blister blight show a strong linear regression pattern, which strongly supports that blister blight intensity decreases with reduced intensity of rainfall, rising temperatures and low humidity (Mur et al., 2015). Although the crop loss changes with the nature of tea varieties and geographical locations, there are no cultivars that are completely resistant to blister blight disease in China, Sri Lanka, India or elsewhere. However, tolerant and susceptible tea varieties showed various degrees of physiological and biochemical changes during the infection of blister blight. Prevention and control of blister blight disease in early stage has not been highly effective due to the lack of suitable biological, chemical and cultural methods. So far, a few fungicides (ie. copper oxychloride, nickel chloride hexahydrate, ergosterol biosynthesis inhibitors) were found to be effective in blister blight disease control (Baby, 2002, Sen et al., 2020). However, for tea quality, the chemical elicitors may be an more eco-friendly approach for disease control, which can improve the innate immunity of tea plants by significantly increasing the level of defense molecule (Sen et al., 2020).

### The development and spread of disease on tea leaf

**Fig. 2:**
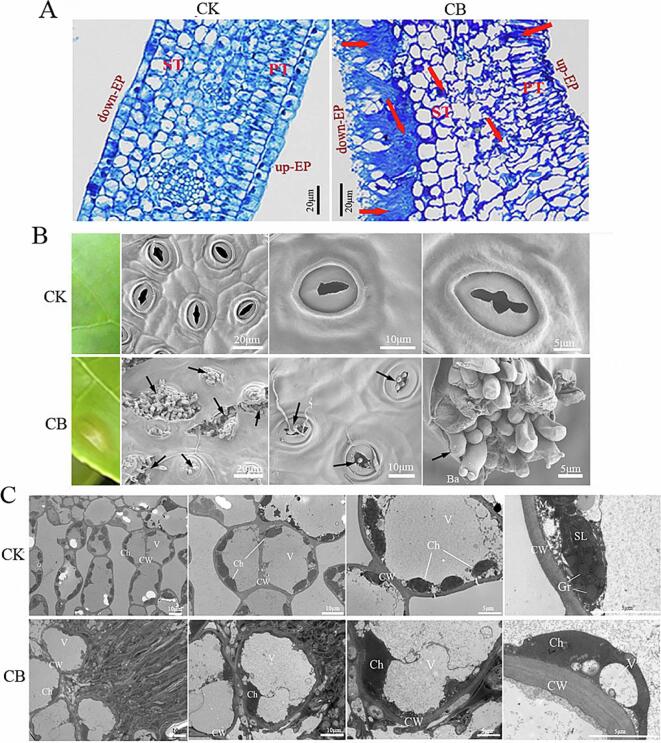
The development and spread of blister blight disease occurring on tea leaves. (A) Tissue structure of tea leaves analyzed by toluidine blue staining. up-EP: up-epidermis; ST: spongy tissue; PT: palisade tissue; down-EP: down-epidermis. The arrows indicate the hyphae of pathogens. bar = 20 μm. (B and C) Scanning electron microscope and transmission electron microscope analysis. CK: healthy tea leaves；CB: infected tea leaves；GC: guard cell；St: stoma；MC: mesophyll cells；Ch: chloroplast; V: vacuole; CW: cell wall; Gr: grana lamella; SL: stroma lamella; M: mitochondria; Ba: basidiospore, basidia. The arrows indicate the basidiospore and hyphae of pathogens. (For interpretation of the references to colour in this figure legend, the reader is referred to the web version of this article.)

The pathogen *Exobasidium* usually infected young tissues and harvestable tea shoots resulting in serious crop loss, and the normal physiological metabolism and growth of tea was negatively affected by the infection of *Exobasidium* (Fig. 1). To reveal the development and spread of the pathogen *E. vexans* on tea leaves, TBS, SEM and TEM analysis were applied. The structure of tea leaves from top to bottom was upper epidermis, palisade tissue, sponge tissue and lower epidermis. As shown in Fig. 2A, in healthy tea leaves, the cells in the upper epidermis were closely arranged without gap between cells; palisade tissue was composed of closely arranged cylindrical cells, which was perpendicular to the upper epidermal cells; below the palisade tissue was sponge tissue that was consisted of loosely arranged parenchyma cells with irregular cell morphology and large cell gap; the lower epidermis was made up of a layer of flat cell, which was densely covered with villi and stomata. Generally, the pathogen of blister blight disease invaded through the stomata of epidermal cells and was stained dark blue (Fig. 2A). With the proliferation of pathogens, basidiospores grew in clusters by consuming nutrients in tea tissue cells (Mur et al., 2015), broke through the epidermis, and a large number of hyphae expanded into the cells of sponge tissue, resulting in the necrosis of tea leaf cells and the destruction of leaf tissue structure (Fig. 2A).

The results of SEM further confirmed that the pathogen causing blister blight disease mainly invaded from the stomata of the lower epidermis of tea leaves, and during infection, the basidiospores born on the top of the basidia propagated in large numbers and distributed throughout the stomata (Fig. 2B). The basidia were clavate, generally bearing two sterigmata, and basidiospores were oval or kidney shaped. In serious cases, the stomatal structure was destroyed, and the guard cells were damaged and deformed, affecting the tissue structure and the development of tea leaves, resulting in the destruction of leaf tissue structure and abnormal growth. In contrast, the lower surface of healthy tea leaves was covered with fine villi, and the stomata were distributed evenly. The cell ultrastructure of healthy tea leaves and infected tea leaves were observed by TEM. As shown in Fig. 2C, the cell wall structure of healthy tea leaf was clear and complete, the organelles in the cell were intact, and the sponge tissue cells were loosely arranged with large intercellular space that was mainly occupied by vacuoles. Chloroplasts were spindle shaped, evenly distributed near the cell wall, with clear structure and membrane coating. The grana thylakoids were stacked neatly, and the stroma lamellae were arranged orderly (Fig. 2C). A small number of mitochondria were distributed around chloroplasts, and the vacuoles were in the center of cells with intact structure. However, the structure of diseased tea leaves was significantly affected by the infection of pathogens. After breaking through the epidermis of tea leaves, the pathogen mainly grew and proliferated in the intercellular space of sponge tissue, and then further infected sponge tissue cells, resulting in the extrusion and deformation of the internal structure of the cells (Fig. 2C). The membrane coating of chloroplasts and grana thylakoids disappeared, and the stroma lamellae were mixed with irregular structure (Fig. 2C). The whole cell was in a dissociated state, numerous of sediments were distributed in the cytoplasm and organelles were stacked (Fig. 2C). In general, after the infection of blister blight disease, the growth and development of tea leaves were negatively affected. Cool temperature (15–25 ℃), higher relative humidity (>80 %), less sunshine (<4h) and long duration of surface wetness (>11 h) are conducive to the infection and spread of basidiospores (Baby, 2002, Mur et al., 2015, Sen et al., 2020).

### Composition and abundance of fungal community on blister blight infected tea leaves

**Fig. 3:**
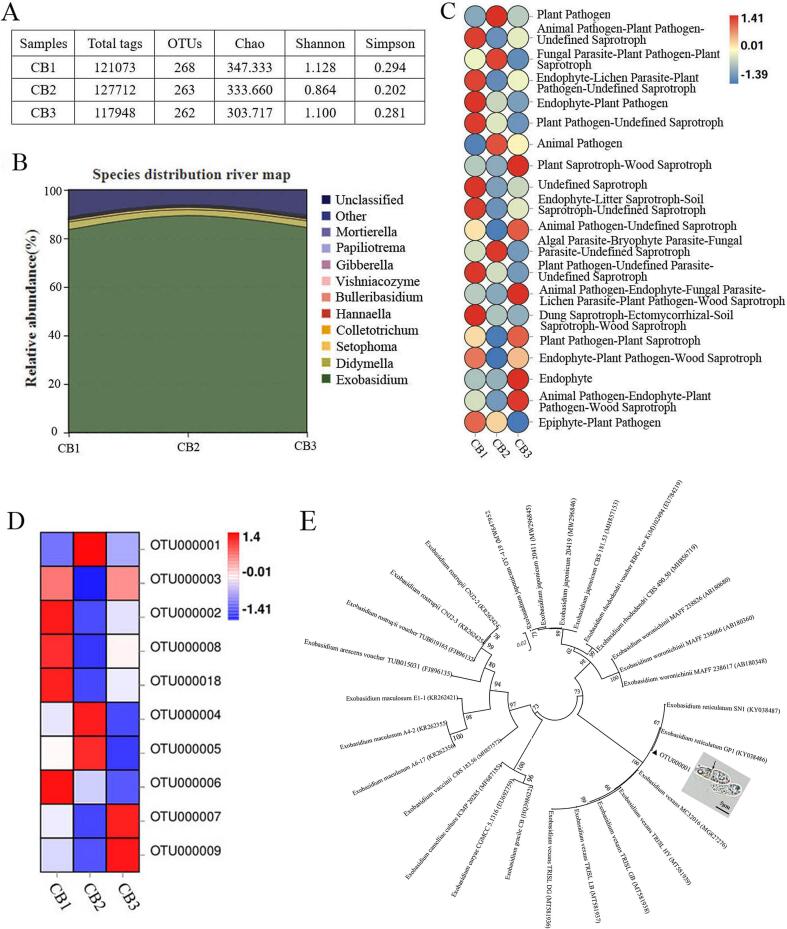
Composition and abundance of fungal community on blister blight infected tea leaves. (A) The alpha diversity index. (B) Species distribution river map. (C) Function distribution heat map. (D) Species abundance. (E) The phylogenetic analysis of the *Exobasidium* species and morphology of basidiospores.

Tea leaves infected with blister blight were generally thickened with powdery coating (Fig. 1). To verify the pathogens on the lesions, the microbial diversity and abundance of tea leaves showing blister blight were detected. After quality filtering, a total of 124,120 sequences (raw tags) were obtained from three samples (ten lesion tissues for each sample, CB1, CB2 and CB3). The alpha diversity index including Shannon, Chao and Simpson index were calculated to determine the species richness and evenness, and there was no significant difference in each index among the three samples. The number of OTUs per sample ranged from 262 to 268 (Fig. 3A). On the base of SILVA taxonomic database, all the gene sequences were classified from phylum to species using analytical program QIIME. Although the distribution of each genus or species varied, the overall fungal composition were similar among samples. In all, the top ten species in mean abundance detected from the diseased lesions were shown in the species distribution river map, and the other known species were classified as others (Fig. 3B). The OTUs that cannot be annotated to defined species were defined as the Unclassified category, and approximately 9.29 % of reads have not been classified. Among the known species, *Exobasidium* was the most abundant fungus accounting for 85.84 % of the total as shown in Fig. 3B. In addition, there were a small amount of *Colletotrichum gloeosporioides* (0.27 %), *Bulleribasidium variabile* (0.11 %) and *Hannaella coprosmae* (0.06 %). Most of the fungi were plant pathogens, followed by saprotrophs and endophytes (Fig. 3C). Our results confirmed the dominant microbial population and abundance of fungal community on tea leaves infected with blister blight disease. However, Barman et al. (2020) found that tea blister blight lesions were colonized by *Nigrospora* and *Pestalotiopsis*, and the two endophytes could also present as potent phytopathogen that could inflict serious damage to tea production, which was different with the results of this study. It may be that the fungal community is remarkably influenced by the surrounding environment and host factors. Among these fungi, the dominant *Exobasidium* is significantly influenced by the environmental factors. Previous studies indicated that a leaf wetness of 11 h was critical for tea blister blight incitation, while temperature more than 32℃ was lethal for the basidiospores, sporulation was prevented at 35℃, and average 3.5 h of sunshine per day over 5 days could significantly reduce the incidence of disease (Visser et al., 1961, Baby, 2002).

Species with abundance greater than 0.1 % in the samples were shown in the Fig. 3D. Among them, OTU000001 was the most abundant fungus which was initially labeled as *Exobasidium* by microbial diversity analysis. For the phylogenetic analysis, the homologous multiple strains of the *Exobasidium* species were collected from GenBank database. The evolutionary tree was constructed by NJ method, and the optimal tree with the sum of branch length is 0.43469523. The phylogenetic analysis based on ITS rRNA region indicated that OTU000001 was grouped together with *E. reticulatum* and also clustered with *E. vexans* in a large clade (Fig. 3E). Blast searches showed that OTU000001 had 99.47–100.00 % identity with *E. reticulatum* strain SN1 (KY038487) and GP1 (KY038486), and 100 % identity with *E. vexans* strain MC32016 (MG827276). Both *E. reticulatum* strains SN1 and GP1 were isolated from blister blight infected tea leaves in India and the paper was unpublished until now. However, for a long time *E. reticulatum* was considered as the causal agent of tea net blister blight disease which had similar disease symptom and morphological characteristics with blister blight disease caused by *E. vexans*. *E. vexans* was the most common pathogen causing tea blister blight disease that had been frequently reported in the tea growing countries worldwide (Sen et al., 2020, Chen and Sun, 2013). Basidiospores were ellipsoid curved, hyaline and initially unicellular, but a distinct septation developed when the basidiospores mature (the black arrow). The size of the basidiospores was 12.7–25.8 × 4.0–6.9 um (Fig. 3E). Although the phylogenetic relationship of *E. vexans* and *E. reticulatum* were closely related, based on the disease symptoms (Fig. 1) and morphological characteristics of *Exobasidium* (Fig. 3E), the dominant pathogen on blister blight lesions was identified as *E. vexans* that systematically placed under *Exobasidium* (Genus), *Exobasidiaceae* (Family), *Exobasidiales* (Order), *Exobasidiomycetes* (Class), *Basidiomycota* (Phylum).

### Sensory evaluation and changes of main non-volatile metabolites in diseased tea

**Fig. 4:**
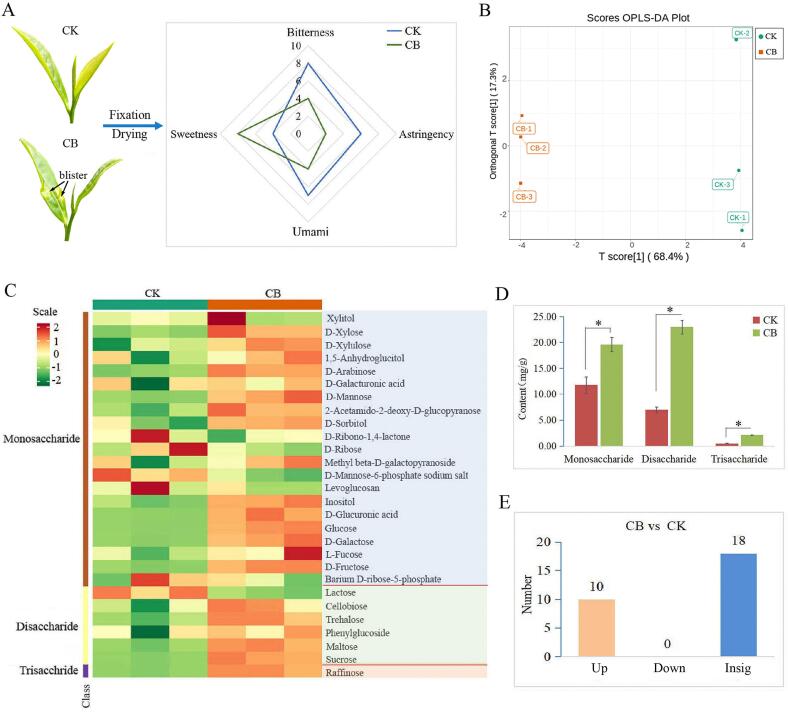
The sensory evaluation and differential sugar metabolites analysis between healthy and diseased tea. (A) Sensory evaluation of tea taste. (B) OPLS-DA scores. (C) The contents and composition of sugar metabolites by metabolomic analysis. (D) The classification of sugar metabolites. Values are mean ± SD and a significant difference is marked as * (P < 0.05). (E) Numbers of the up- and down-regulated sugar metabolites between healthy and diseased tea.

Blister blight disease plays an important role in the growth and development of tea trees, which directly influences the tea quality and flavor. So far, the molecular and biochemistry basis of blister blight disease resistance in tea plants have been well studied (Nisha et al., 2018, Premkumar et al., 2008, Mur et al., 2015; Jayaswall et al., 2015), however, there are limited information on the tea quality and flavor influenced by this disease. Taste is the most important factor in determining the quality and flavor of tea. Sensory evaluation results showed that diseased tea leaves had strong sweetness with weak bitter, astringent and umami taste compared with healthy tea leaves (Fig. 4A), which is not in accordance with the previous results that diseased tea leaves showed obvious bitter taste (Guo et al., 2005, Jayaswall et al., 2016). To reveal the sensory difference, the taste related metabolites between healthy and diseased tea leaves were investigated. OPLS-DA was performed to distinguish the significantly differential metabolites between CK and CB based on the principle of VIP ≥ 1, P value < 0.05 and fold change ≥ 2 or ≤ 0.5 (Fig. 4B). As the main water-soluble carbohydrate in tea, soluble sugar significantly contributes to sweetness of tea (Rolland et al., 2006). The sugars in tea leaves mainly include water-soluble monosaccharides and disaccharides, water-insoluble polysaccharides and a small amount of other saccharides. In this study, total 28 sugars were detected in CK and CB, both including 21 monosaccharides, 6 disaccharides, and 1 trisaccharide (Fig. 4C). The content of total sugars in infected tea leaves was remarkably increased by 131.96 % (increased from 19.26 to 44.66 mg/g) (Fig. 4C), indicating that the accumulation of total sugars is significantly induced by the infection of blister blight disease and the change is positively associated with the enhanced sweet taste of tea. Sugars not only play important roles in the development of tea plant, yield formation and stress response mainly by producing multiple sugars to fuel growth and synthesize essential compounds, but also can be used as carbon sources to provide material basis for the growth of heterotrophic microorganisms (Lastdrager et al., 2014, Rolland et al., 2006).

21 monosaccharides, 6 disaccharides and 1 trisaccharide accounted for 61.26 %, 36.22 % and 2.52 % of the total sugars in CK, respectively, while in CB, the three groups accounted for 43.93 %, 61.43 % and 4.64 %, respectively (Fig. 4D), showing a significant change by the infection of blister blight disease. Significantly changed metabolites between healthy and diseased tea were determined by VIP ≥ 1, P value < 0.05 and fold change ≥ 2 or ≤ 0.5. Among the 28 sugars, 10 up-regulated sugars (≥2 fold change) were detected in CB compared with healthy tea leaves, while the other 18 sugars showed no significant changes (0.5 < fold change < 2) (Fig. 4E). Among the 10 up-regulated sugars, sucrose and maltose belonging to disaccharide accounted for 50.40 % of the total sugars, 7 kinds of monosaccharides including d-xylose, d-arabinose, d-mannose, d-glucuronic acid, glucose, d-galactose and d-fructose only accounted for 15.49 %, and the other 1 trisaccharide (raffinose) accounted for 4.64 % in CB, indicating that monosaccharide and disaccharide were the most abundant differential soluble sugars in diseased tea, which may contribute to the enhanced sweet taste of diseased tea. However, Pius et al. (1998) found that sucrose and glucose contents were significantly decreased in the blistered lesions, and the content of fructose was remarkably increased during the initiation of sporulation and remained constant up to the end of sporulation in non-blistered and blistered regions. The decrease of the sugar accumulation in diseased tea leaves may be due to the increase in the utilization or consumption rate by the pathogen as material basis during the infection process. However, in this study, most of the soluble sugars including sucrose, glucose and fructose detected in raw materials (one bud and two leaves) were all significantly increased (Fig. 4C), which may be due to the different infection stages, tea varieties, etc.

### The metabolic pathway enrichment analysis

**Fig. 5:**
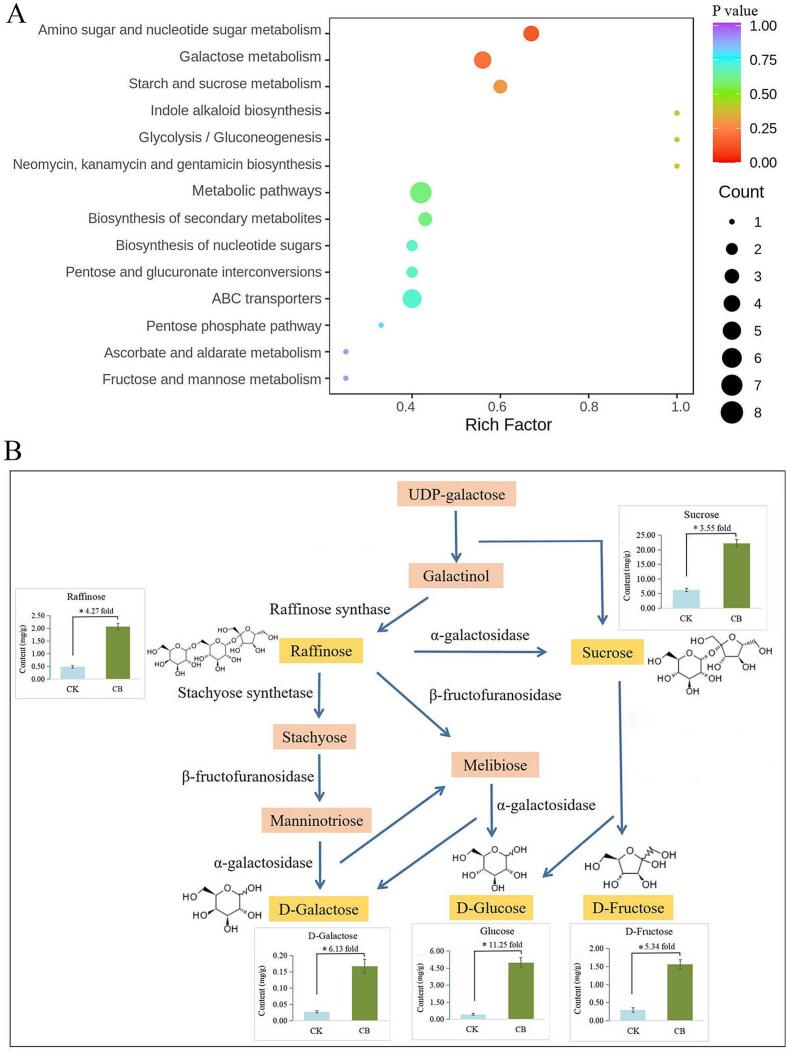
The KEGG enrichment analysis of sugars metabolic pathways (A) and the main sugar biosynthetic pathway (B). The content and fold change of main sugar metabolites were shown as the means ± SD (n = 3). A significant difference is marked as * (P < 0.05).

**Fig. 6:**
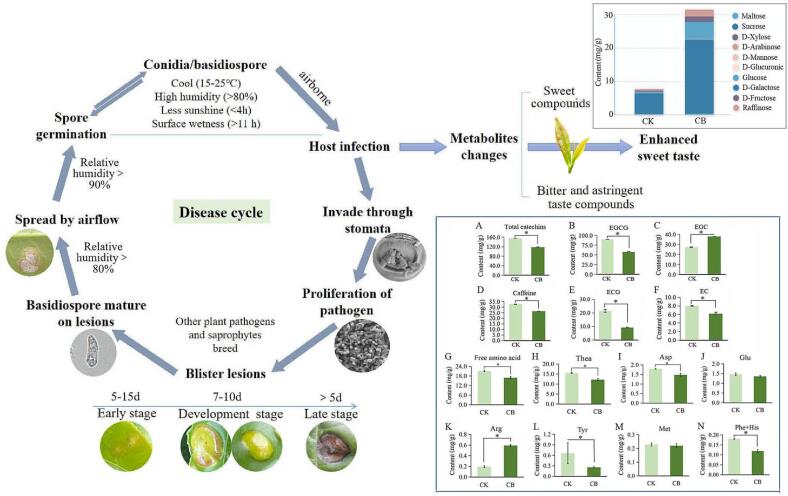
Overview of the disease cycle of blister blight on tea plants and the influence on tea quality and flavor. (A) Total catechins. (B) EGCG ((−)-epigallocatechin gallate). (C) EGC ((−)-epigallocatechin). (D) Caffeine. (E) ECG ((−)-epicatechin gallate). (F) EC ((−)-epicatechin). (G) Free amino acid. (H) Thea (Theanine). (I) Asp (Aspartic acid). (J) Glu (Glutamic acid). (K) Arg (Arginine). (L) Tyr (Tyrosine). (M) Met (Methionine). (N) Phe (Phenylalanine) and His (Histidine). The content of metabolites were shown as the means ± SD (n = 3). A significant difference is marked as * (P < 0.05).

Based on the KEGG functional annotations and pathway enrichment analysis, significantly differential soluble sugars including monosaccharide, disaccharide and trisaccharide were involved in 14 metabolic pathways (Fig. 5A). Among the metabolisms, galactose metabolism, amino sugar and nucleotide sugar metabolism, and starch and sucrose metabolism were the significant enrichment metabolisms involving in the response to blister blight disease (Fig. 5A). The greater the rich factor, the more significant the enrichment. The larger size of the dot represents the more differential metabolites enriched in the pathway. Moreover, five sugars (sucrose, d-glucose, d-galactose, d-fructose and raffinose) that significantly increased in diseased tea leaves were mainly involved in galactose metabolism (Fig. 5B). Sucrose, d-glucose, d-galactose, d-fructose and raffinose accounting for 69.63 % of the total sugars were the abundant differential soluble sugars showing 3.55, 11.25, 6.13, 5.34 and 4.27 fold higher in CB than those in CK, respectively (Fig. 5B). Although the fold change of sucrose is much lower than the other four sugars, sucrose was the most abundant soluble sugar in tea with the content increased by 255.09 %, accounting for 49.93 % of the total sugars in CB (Fig. 5B). Sucrose was converted from the raffinose family of oligosaccharides by α-galactosidase, and could be used as substrates for the synthesis of d-glucose and d-fructose. Glucose was the second abundant differential sugar which increased from 0.44 to 4.99 mg/g with the highest fold change in CB, followed by raffinose, d-fructose and d-galactose with the content of 2.07, 1.56 and 0.17 mg/g, respectively (Fig. 6B). Raffinose was converted into d-glucose and d-galactose through several intermediate products. The result indicated that the five significantly up-regulated metabolites in sugar metabolism are significantly induced by the infection of blister blight disease which plays a vital role in the tea growth. At present, there were no reports on related sugar metabolism pathway analysis involving in disease infection on tea, however, previous studies found that carbohydrate metabolism (starch and sucrose, fructose and mannose) can be activated by the damage of tea geometrid (Wang, 2018), and sugar metabolism is closely related to the response of tea plants to low temperature stress (Yue, 2015).

### The influence of blister blight disease on tea quality

Generally, besides soluble sugars, the sweetness characteristic of tea is affected by the comprehensive effect of various kinds of metabolites, especially caffeine (bitterness), catechins (bitterness and astringency) and Thea (umami taste) (Cao et al., 2021, Cho et al., 2017, Wang et al., 2010). The contents of catechins (Fig. 6A) and caffeine (Fig. 6D) in blister blight infected tea leaves were all decreased by 23.9 % and 19.22 %, respectively. Gulati et al. (1999) also showed that the significant decrease in catechins was highly related to high disease severity. During the infection of the pathogen *E. vexans* in tea, catechins can be polymerisated to oligomeric proanthocyanidins and 2,3-cis isomerisation (Punyasiri et al., 2004). As important components of catechins, the contents of epigallocatechin gallate (EGCG) (Fig. 6B), epicatechin gallate (ECG) (Fig. 6E) and EC (Fig. 6F) were all remarkably decreased by 35.36 %, 57.91 % and 22.68 % respectively, while the content of EGC (Fig. 6C) was significantly increased by 39.15 %, indicating that EGC was induced by the infection of blister blight disease. Total free amino acids content in CB was significantly decreased by 24.67 % compared with that in CK (Fig. 6G), which was consistent with the results of Premkumar et al. (2008). The decrease of chemical constituents may be due to the degradation by certain secreted metabolites or utilization by the pathogen. Amino acids including the most abundant Thea, glutamic acid (Glu) and aspartic acid (Asp) were thought to be closely associated with the umami taste of tea (Cheng et al., 2017), and the contents of Thea (Fig. 6H), Asp (Fig. 6I) and Glu (Fig. 6J) were all significantly decreased in CB compared with that in CK. However, some amino acids such as arginine (Arg) (Fig. 6K), tyrosine (Tyr) (Fig. 6L) methionine (Met) (Fig. 6M), histidine (His) and phenylalanine (Phe) (Fig. 6N) were proved to have bitter taste (Kirimura et al., 1969), and the five amino acids in this study were detected in low contents (<1.0 mg/g). The contents of Tyr, Met and His + Phe were all significantly decreased in CB compared with that in CK, while Arg was significantly enhanced in infected tea leaves (Fig. 6). The increase in the content of Arg may be attributed to the degradation of protein. In summary, the development and spread of blister blight on tea are closely associated with the climatic conditions such as temperature, humidity and sunshine, which significantly influence the metabolites of tea, resulting the quality and flavor change (Fig. 6). The accumulation of caffeine, catechins and its main derivatives (EGCG, ECG and EC), and amino acids and its main components (Thea, Glu and Asp) were all significantly decreased in blister blight infected tea leaves, while EGC, Arg and soluble sugars content were significantly increased (Fig. 6). Therefore, under the suitable environmental conditions (cool temperature, higher relative humidity, etc.), after the infection of *E. vexans*, the metabolites showing sweet taste in the diseased leaves were significantly increased, while the most of the metabolites showing bitter and astringent taste were obviously reduced, indicating that the changes of these metabolites may be of great significance to enhance the sweet taste of tea.

## Conclusions

Blister blight is a low temperature and high humidity type disease, which not only endangers the growth and development of tea trees, but also affects tea quality and flavor. The disease symptoms, the spread of disease on tea leaves, and the composition and abundance of fungal community on blister tissues were fully described, which greatly advances the understanding of the influence of blister blight disease on tea plants and can provide valuable information to develop an integrated management strategy for the control of blister blight disease. Microbial diversity analysis showed *Exobasidium* was the most abundant fungus (85.84 %) in blister tissues. Tea quality is affected by the comprehensive effect of various non-volatile substances which is significantly changed by disease infection. The main monosaccharides, disaccharide and trisaccharide in sugar metabolism were significantly induced by the infection of blister blight, and sucrose, d-glucose, d-galactose, d-fructose and raffinose (accounting for 69.63 % of the total sugars) were the abundant differential sugars showing 3.55, 11.25, 6.13, 5.34 and 4.27 fold higher in infected tea leaves, respectively. Furthermore, the bitter taste related metabolites including caffeine, catechins (EGCG, ECG and EC) and some amino acids (Thea, Asp and Glu) were significantly decreased, which also greatly contributes to the enhanced sweet taste of diseased tea.

## Declaration of competing interest

The authors declare that they have no known competing financial interests or personal relationships that could have appeared to influence the work reported in this paper.

## Data Availability

Data will be made available on request.

## References

[b0005] Baby U.I. (2002). An overview of blister blight disease of tea and its control. Journal of Plantation Crops.

[b0010] Barman A., Nath A., Thakur D. (2020). Identification and characterization of fungi associated with blister blight lesions of tea (Camellia sinensis L. Kuntze) isolated from Meghalaya, India. Microbiological Research.

[b0015] Cao Q.Q., Wang F., Wang J.Q., Chen J.X., Yin J.F., Li L., Y., q. (2021). Effects of brewing water on the sensory attributes and physicochemical properties of tea infusions. Food Chemistry.

[b0020] Caporaso J., Kuczynski J., Stombaugh G., Bittinger K., Bushman F.D., Costello E.K., Gordon J.I. (2010). QIIME allows analysis of high-throughput community sequencing data. Nature Methods.

[b0025] Chen Y.J., Yang J., Meng Q., Tong H.R. (2023). Non-volatile metabolites profiling analysis reveals the tea flavor of “Zijuan” in different tea plantations. Food Chemistry.

[b0030] Chen Y.Y., Fu X.M., Mei X., Zhou Y., Cheng S.H., Zeng L.T., Yang Z.Y. (2017). Proteolysis of chloroplast proteins is responsible for accumulation of amino acids in dark-treated tea (Camellia sinensis) leaves. Journal of Proteomics.

[b0035] Chen Z.M., Sun X.L. (2013).

[b0040] Cheng S.H., Fu X.M., Wang X.Q., Liao Y.Y., Zeng L.T., Dong F., Yang Z.Y. (2017). Studies on the biochemical formation pathway of the amino acid L-theanine in tea (Camellia sinensis) and other plants. Journal of Agricultural and food Chemistry.

[b0045] Cho J.Y., Mizutani M., Shimizu B., Kinoshita T., Ogura M., Tokoro K., Sakata K. (2017). Chemical profiling and gene expression profiling during the manufacturing process of Taiwan oolong tea ″Oriental Beauty″. Bioscience, Biotechnology and Biochemistry.

[b0050] De Silva R.L., Murugiah S., Saravanapavan T.V. (1992). Blister blight disease in Sri Lankan tea plantations. Tea Quar.

[b0055] Gong S., Zhao Y., Lu C. (2018).

[b0060] Gulati A., Gulati A., Ravindranath S.D., Gupta A.K. (1999). Variation in chemical composition and quality of tea (Camellia sinensis) with increasing blister blight (Exobasidium vexans) severity. Mycological Research.

[b0065] Guo C.Q., Wang W.L., Wu N. (2005). Culture of Exobasidium gracile (Shirai) Syd and its stimulating effects. Journal of Jishou University (natural science edition).

[b0070] Jayaswall, K., Mahajan, P., Singh,G., Parmar, R., Seth, R., Raina, A., ... Sharma, R.K. (2016). Transcriptome analysis reveals candidate genes involved in blister blight defense in tea (*Camellia sinensis* (L) Kuntze). *Scientific Reports*, *6*, Article 30412. 10.1038/srep30412 PMC4964330 27465480

[b0075] Kirimura J., Shimizu A., Kimizuka A., Ninomiya T., Katsuya N. (1969). Contribution of peptides and amino acids to the taste of foods. Journal of Agricultural and Food Chemistry.

[b0080] Lastdrager J., Hanson J., Smeekens S. (2014). Sugar signals and the control of plant growth and development. The Journal of Experimental Botany.

[b0085] Liu L. (2017). Occurrence, prevalence and comprehensive control of tea blister blight disease in tea gardens in Huoshan. Modern Agricultural Science and Technology.

[b0090] Liu R., Yao Y.X., Wang S.Y., Li Y.B. (2021). Investigation on tea blister blight disease in tea gardens of Hongmin, Duyun city. Fujian Tea.

[b0095] Mei X., Liu X.Y., Zhou Y., Wang X.Q., Zeng L.T., Fu X.M., Yang Z.Y. (2017). Formation and emission of linalool in tea (Camellia sinensis) leaves infested by tea green leafhopper (Empoasca (Matsumurasca) onukii Matsuda). Food Chemistry.

[b0100] Mur L.A.J., Hauck B., Winters A., Heald J., Lloyd A.J., Chakraborty U., Chakraborty B.N. (2015). The development of tea blister caused by Exobasidium vexans in tea (Camellia sinensis) correlates with the reduced accumulation of some antimicrobial metabolites and the defence signals salicylic and jasmonic acids. Plant Pathology.

[b0105] Nisha S.N., Prabu G., Mandal A.K.A. (2018). Biochemical and molecular studies on the resistance mechanisms in tea [Camellia sinensis (L.) O. Kuntze] against blister blight disease. Physiology and Molecular Biology of Plants.

[b0110] Petch T. (1923).

[b0115] Pius P.K., Krishnamurthy K.V., Nelson R. (1998). Changes in saccharide metabolism induced by infection of *Camellia sinensis* by *Exobasidium vexans*. Biology Plant.

[b0120] Ponmurugan P., Manjukarunambika K., Gnanamangai B.M. (2016). Impact of various foliar diseases on the biochemical, volatile and quality constituents of green and black teas. Australasian Plant Pathology.

[b0125] Premkumar R., Ponmurugan P., Manian S. (2008). Growth and photosynthetic and biochemical responses of tea cultivars to blister blight infection. Photosynthetica.

[b0130] Punyasiri P.A.N., Tanner G.J., Abeysinghe S.B., Kumar V., Campbell P.M., Pradeepa N.H.L. (2004). *Exobasidium vexans* infection of *Camellia sinensis* increased 2,3-cis isomerization and gallate esterification of proanthocyanidins. Phytochemisty.

[b0135] Punyasiri P.A., Abeysinghe S.B., Kumar V. (2005). Preformed and induced chemical resistance of tea leaf against *Exobasidium vexans* infection. Journal of Chemical Ecology.

[b0140] Radhakrishnan B., Baby U.I. (2004). Economic threshold level for blister blight of tea. Indian Phytopathology.

[b0145] Ran L.X., Xiao X., Ying L.Q., Liang M.Z., Xia R., Rao B.Y., Wang G.Q. (2021). Time and space of *Exobasidium vexans* Massee in Yunnan tea growing area. Southwest China Journal of Agricultural Sciences.

[b0150] Rolland F., Baena-Gonzalez E., Sheen J. (2006). Sugar sensing and signaling in plants: Conserved and novel mechanisms. Annual Review of Plant Biology.

[b0155] Sen S., Rai M., Das D., Chandra S., Acharya K. (2020). Blister blight a threatened problem in tea industry: A review. Journal of King Saud University -Science.

[b0160] Shi M.G., Li Y.Q., Shi L., Duan K.P., Long Y.J. (2016). Outbreak causes and control measures of tea blister blight disease in Jishou City. Modern Agricultural Science and Technology.

[b0165] Sinniah G.D., Wasantha K.L., Karunajeewa D.G.N.P., Ranatunga M.A.B. (2016). Development of an assessment key and techniques for field screening of tea (Camellia sinensis L.) cultivars for resistance to blister blight. Crop Protection.

[b0170] Sharma J., Bhardwaj V.K., Singh R., Rajendran V., Purohit R., Kumar S. (2021). An in-silico evaluation of different bioactive molecules of tea for their inhibition potency against non structural protein-15 of SARS-CoV-2. Food Chemistry.

[b0175] Visser T., Shanmuganathan N., Sabanegam J.V. (1961). The influence of sunshine and rain on tea blister blight *Exobasidium vexans* Massee in Ceylon. Annals of Applied Biology.

[b0180] Wang L., Xu R.J., Hu B., Li W., Sun Y., Tu Y.Y., Zeng X.X. (2010). Analysis of free amino acids in Chinese teas and flower of tea plant by high performance liquid chromatography combined with solid-phase extraction. Food Chemistry.

[b0185] Wang R., Xiao W.P., Zheng S., Luo Q.L., Wei Q., Deng Z.G. (2013). Investigation and control technology of tea blister blight disease in Duyun City. Agricultural Technology Service.

[b0190] Wang W. (2018).

[b0195] Yue C. (2015).

[b0200] Zeng L.T., Watanabe N., Yang Z.Y. (2019). Understanding the biosyntheses and stress response mechanisms of aroma compounds in tea (Camellia sinensis) to safely and effectively improve tea aroma. Critical Reviews in Food Science and Nutrition.

