# Supplementary material for: Effect of blister blight disease caused by Exobasidium on tea quality
Source: Food Chem X. 2023 Dec 18;21:101077. doi: 10.1016/j.fochx.2023.101077 (PMC10788223; doi:10.1016/j.fochx.2023.101077)
Supplement: Supplementary data 1 [file mmc1.docx]

Table S1 The occurrence and influence of blister blight disease on tea worldwide

| **Location** | **Climate type** | **Main varieties** | **Annual average temperature** | **Altitude** | **Relative humidity** | **Climate characteristics** | **Peak incidence** | **Disease incidence** | **References** |
| --- | --- | --- | --- | --- | --- | --- | --- | --- | --- |
| Quxian/ Tianquan /Dujiangyan, Sichuan province, China | Subtropical monsoon climate | Sichuan middle- and small-leaf tea tree populations, Fujian tea tree populations | 13 - 20.8℃ | 800 - 1420m | 80 - 95% | In 2019 and during 1980 to 1990，in the middle of April, 13 - 16℃, tea blister blight began to appear; with the increase of temperature and humidity, the disease aggravated; after November, the temperature fell to 8.0 - 13.3℃, resulting in the disappearance of disease. With the increase of altitude, the incidence increased correspondingly. | April - May, Late August - September | 6.56 - 35.04% | In this study; Jiang et al., 1993 |
| Yunnan province, China | Subtropical monsoon climate | Zijuan, Yunkang 10, Yunkang 22, Pujing 1, Foxiang 4, Qingshui 3, Xiangguiyinhao, Fengqing large-leaf populations | - | 1096 - 1884m | - | In 2008 and 2019, early rainy season, long duration and heavy rainfall, high humidity. Tea blister blight began to appear from late June to early August in different tea growing areas; the disease was more serious in tea gardens with high shade. | August - October | 14.29 - 65.0 % | Shan and Tang 2010; Ran et al., 2021 |
| Duyun, Guizhou province, China | Subtropical monsoon climate | - | - | 660 - 1460m | - | In 2010 and 2011, tea blister blight began to appear in the first and middle of May with dense fog; the degree of disease varied with the altitude and season; summer tea was the most affected throughout the year. | June - July, September | 5.95 - 53.81% | Wang et al., 2013; Liu et al., 2021 |
| Wuzhishan, Hainan province, China | Tropicl ocean monsoon climate | large-leaf tea tree populations | 22.4℃ | 1800m | - | In 2019-2020, tea blister blight began to appear in late February, with heavy fog in the morning and evening; from the early of December, disease disappeared with the reduction of rainfall and temperature. | March - November | >85% | Ning et al., 2020 |
| Lishui, Zhejiang province, China | Subtropical monsoon climate | Yingshuang, Longjin43, Anjibaicha, Zhongcha108, Wuniuzao, longjinchangye varieties | 19.2 - 23.8℃ | 260-520m | - | In 2010, rainfall was closely related to the occurrence of blister blight disease from April to June and August to October every year；tea gardens with 60% shade was more conducive to the occurrence of disease. | April - June,  August - October | 12.33 - 70.33% | Wu et al., 2013 |
| Enshi/Yichang/Shiyan/Yidu, Hubei province, China | Subtropical monsoon climate | Wuniuzao, Fudingdabaicha, Echa 1 varieties | 16.7℃ | > 500m | >80% | During 2013-2014 and in 2018,  in May, with the increase of temperature and rainfall, pale yellow spots appeared on tender tea shoots; in June, with the increase of the temperature and humidity (＞80%), the incidence reached 15-35%; from July to August, the rainfall was the most and a large number of basidiospores were released, reaching the peak of the disease; during September to October, with the the decrease of temperature and humidity, the basidiospores were difficult to release; in November, disease disappeared when the average temperature was lower than 15 ℃. | June - September | 12.5 - 56.3% | Tan et al., 2015; Liao et al., 2019 |
| Huoshan, Anhui province, China | Transition zone between subtropical humid monsoon climate and temperate semi humid monsoon climate | Wuniuzao, Shuchazao, Jingjizao, Longjing varieties | 15℃ | - | 80 - 90% | In 2016, the climate was warm and humid with heavy rainfall and fog, and the annual precipitation was about 1500 mm, concentrating in May to July; the frost free period was about 230d. | May - July, September - October | - | Liu et al., 2017 |
| Jishou, Hunan province, China | Subtropical monsoon humid climate | - | 20.3 - 22.7℃ | 200 - 450m | 76 - 87% | In 2015, the tea gardens located in the mountain canyon had a forest coverage rate of 100% and heavy fog and dew in the morning and evening. | March - May, August - October | 10.0 - 58.2% | Shi et al., 2016 |
| Cuona, Tibet Autonomous Region, China | Subtropical mountain humid sub-humid monsoon climate | - | 15 - 20℃ | 2300m | >85% | The alpine tea gardens were damp, overgrown with weeds and less sunshine. | July - August | - | Jin et al., 2014 |
| Sri Lanka | Tropical monsoon climate | - | 15.1 - 23.3℃ | 600 - 1200m, >1200m | 71.9 - 95.9% | In 2012 and 2013, tea gardens at medium to high altitudes were susceptible to blister blight during the southwest and northeast monsoons each year. Higher rainfall and fewer sunshine hours in 2013 resulted in higher disease severity almost in all cultivars, while less rainfall and more sunshine hours in 2012 resulted in less disease almost in all cultivars. | May - September, October - December | 0 - 30% | Sinniah et al., 2016 |
| Tamil Nadu, India | Tropical monsoon climate | Assamica tea cultivars, TRI 2043, TRI 777, DT1, TRI 3072, DN, TRI 4079, TRI 3020, TRI 4054, TRI 2025 | 12 - 30℃ | 2200m | 85 - 95% | In 2017, there was drastic fluctuation in the weather pattern with mean rainfall of c. 1500 mm per annum. High temperature all year round, the dry and rainy season was obvious, the precipitation was concentrated in the rainy season, and the humidity in rainy season was high. | June - December | 6.3 - 14.5% | Sowndhararajan et al., 2013 |
| Darjeeling, India | Tropical monsoon climate | Assamica variety TV-9 | - | 950 - 1830m |  | In 2014, the high temperature throughout the year, the dry season and the rainy season were distinct, the precipitation was concentrated in the rainy season, and the amount of precipitation was large. | June - September | - | Mur et al., 2015 |
